# Metabolomics and transcriptomics reveal the effect of hetero-chitooligosaccharides in promoting growth of *Brassica napus*

**DOI:** 10.1038/s41598-022-25850-7

**Published:** 2022-12-08

**Authors:** Chao Tang, Yang Zhai, Zhuo Wang, Xin Zhao, Chen Yang, Yong Zhao, Liang-bin Zeng, De-yong Zhang

**Affiliations:** 1 College of Plant Protection, Hunan Agricultural University, No. 1, Nongda Road, Furong District, Changsha City, 410208 Hunan Province China; 2 Institute of Bast Fiber Crops, Chinese Academy of Agricultural Sciences, No. 348, Xianjiahu West Road, Yuelu District, Changsha, 410205 Hunan Province China; 3 State Key Laboratory of Biochemical Engineering, Institute of Process Engineering, Chinese Academy of Sciences, Beijing, 100190 China; 4 ZhongkeRunxin (Suzhou) Biotechnology Co., Ltd., Suzhou, 215152 Jiangsu China

**Keywords:** Molecular biology, Physiology

## Abstract

The hetero-chitooligosaccharide (HTCOS) is a naturally occurring biopolymer in the exoskeleton of crustaceans and insects. Although some studies have been carried out on HTCOS in inducing plant resistance and promoting growth, the molecular mechanism of HTCOS in plants is not clear. In this study, an integrated analysis of metabolomics and transcriptomics was performed to analyze the response of *Brassica napus* to hetero-chitooligosaccharides treatment. The levels of 26 metabolites in *B. napus* were significantly changed under the HTCOS treatment. Amongst these metabolites, 9 metabolites were significantly up-regulated, including pentonic acid, indole-3-acetate, and γ-aminobutyric acid. Transcriptome data showed that there were 817 significantly up-regulated genes and 1064 significantly down-regulated genes in *B. napus* under the HTCOS treatment. Interestingly, the indole-3-acetate (IAA) content under the HTCOS treatment was about five times higher than that under the control condition. Moreover, four genes related to plant hormone signal transduction, three AUX/IAA genes, and one ARF gene, were significantly up-regulated under the HTCOS treatment. Furthermore, the plant height, branching number, and biomass of *B. napus* under the HTCOS treatment were significantly increased compared to that in the control condition. This evidence indicated that the HTCOS treatment contributed to accumulating the content of plant hormone IAA in the *B. napus*, up-regulating the expression of key genes in the signaling pathway of plant growth and improving the agronomic traits of *B. napus*.

## Introduction

Hetero-chitooligosaccharides (HTCOS), chitooligosaccharides (COS), N-acetyl-chitooligosaccharides (NACOS), and other oligosaccharides derived from chitin, are obtained by enzymatic preparation of chitin, a naturally occurring biopolymer in the exoskeleton of crustaceans and insects. These oligosaccharides are all composed of *N*-acetylglucosamine and its deacetylation product, glucosamine, with a degree of polymerization < 20 and an average molecular weight < 3.9 kDa, differing only in the ratio of the glucosamine and GlcNAc (Degree of deacetylation)^1,2^. These low molecular weight products have good water solubility, easy absorption, high bioactivity, and are environmental-friendly^3–5^.

Chitin-derived oligosaccharides, such as COS and NACOS have good plant immune-inducing activity. A variety of bio-pesticide products containing COS as the major ingredient has been registered in China and widely used in agricultural production^6^. The acetyl group on the monosaccharide is essential for binding the oligosaccharide to the receptor. The COS had a degree of deacetylation at around 50–70% indicating strong plant immune-modulating activity^7^. Also, higher polymerization levels result in greater activity of Chitin-derived oligosaccharides.

Numerous studies have shown that COS can not only induce an increase in the expression level of endogenous plant hormones such as indoleacetic acid^8,9^, gibberellin, and salicylic acid in plants but also cause an increase in the activity of defense enzymes such as polyphenol oxidase and peroxidase in plants^10,11^. COS is an effective immune exciton in plants^12^, enabling the induction of resistance to cold, disease, and insects^13^. Recently, HTCOS oligosaccharide products with 50–80% deacetylation, have become the latest research direction for chitin-derived oligosaccharides. HTCOS have a sufficient number of acetyl groups contributing to the binding of receptors on the plant surface, thus effectively activating the plant immune system. On the other hand, their positive charge can also play an active role, while still having good water solubility at a high degree of polymerization (DP > 8), unlike NACOS. In addition, the degree and pattern of deacetylation of HTCOS is closer to the fungal cell wall chitin/chitosan, the natural plant excitons, which may also be the structural basis for their excellent plant immune-inducing activity.

Oilseed rape is one of China's important oil crops. About 50% of edible vegetable oil is from oilseed rape, in the domestic edible vegetable oil occupies an important position, cultivating oilseed rape seedlings is to seize the key foundation of high and stable yields. In rape cultivation, COS is widely used for seed coating^14^ to promote the growth of rape seedlings, and has been shown to induce resistance to *Sclerotinia sclerotiorum*, a major disease of rape^15^, as well as to improve photosynthesis, frost and drought resistance in rape^16^, and to alleviate high salt damage^17^. HTCOS also promotes the growth and development of oilseed rape, improves agronomic traits such as branch number and biomass, and induces resistance to the small rape moth. This paper analyses the response of kale-type oilseed rape to HTCOS through a combination of transcriptomic and metabolomic analyses to investigate changes in key genes in the growth signal transduction pathway in oilseed rape under HTCOS treatment.

## Results

### Metabolic changes in *Brassica napus* under the HTCOS treatment

**Figure 1:**
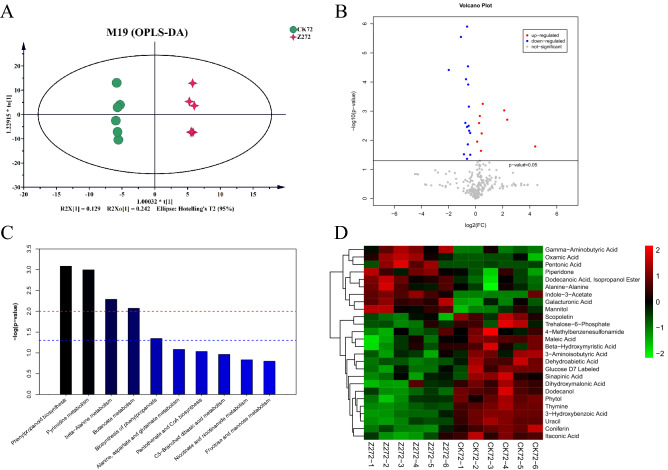
The changes of metabolites in *B. napus* between control (CK72) and HT-COS (Z272) treatments. (**A**) The orthogonal partial least squares discriminant analysis in metabolite contents. (**B**) The volcano plot of significantly differentiated metabolite contents. (**C**) The gene functional enrichment of significantly up-regulated metabolites under the DA treatment. (**D**) The heat map of metabolites changes.

To assess the response of *B. napus* to the HTCOS treatment, we analyzed the difference of metabolites in *B. napus* leaves. The orthogonal partial least squares discriminant analysis showed significant differences in metabolite content between HTCOS treatment and control (Fig. 1A). The level of 26 metabolites (9.89% of total metabolites) was significantly changed (*p*-value < 0.05) in response to HTCOS treatment, including nine up-regulated metabolites and seventeen down-regulated ones (Fig. 1B and Supplementary Table 1). Among the 26 different metabolites, the difference in metabolite content between the treatment group and the control group was 1.87 times on average, ranging from 0.25 to 21.12 (Supplementary Table 1).

Functional annotation based on the KEGG database indicated that a total of 18 changed metabolites were enriched in 15 KEGG pathways, related to phenylpropanoid biosynthesis, pyrimidine metabolism, beta-alanine metabolism, butanoate metabolism, hormones metabolic pathway, and so on (Fig. 1C,D). Especially, the contents of pentonic acid, indole-3-acetate (IAA), and gamma-aminobutyric acid under the HTCOS treatment were 21.1, 5.0, and 4.3 times higher than that in the control condition, respectively.

### Effects of gene expression in *Brassica napus* under the HTCOS treatment

A total of 95,225,228 (~ 13.8 Gb) and 94,206,911 (~ 13.6 Gb) transcriptome reads on average were generated for three biological replicates of *Brassica napus* samples under the HT-COS or blank treatment respectively (Table 1). About 95% of reads could align to the reference genome of *B. napus*, including 62.5% of multiple mapped and 33.2% of uniquely mapped. Approximately 67.6% and 67.7% of total genes were aligned by the transcriptome data under the two conditions, respectively.

**Table 1:** Data statistics after transcriptome sequencing filtered for *B. napus* experimental treatments.

Sample	Raw reads	Raw bases	Clean reads	Clean bases	Valid bases (%)	Q30 (%)	GC (%)
CK72-7	98.00M	14.70G	94.44M	13.72G	93.31	93.09	42.62
CK72-8	99.03M	14.85G	95.03M	13.83G	93.13	92.80	42.73
CK72-9	99.61M	14.94G	96.21M	13.97G	93.47	93.39	42.85
Z272-7	99.81M	14.97G	94.14M	13.58G	90.72	91.88	43.18
Z272-8	98.47M	14.77G	93.59M	13.50G	91.42	92.43	43.17
Z272-9	99.49M	14.92G	94.89M	13.72G	91.96	92.71	42.94

**Figure 2:**
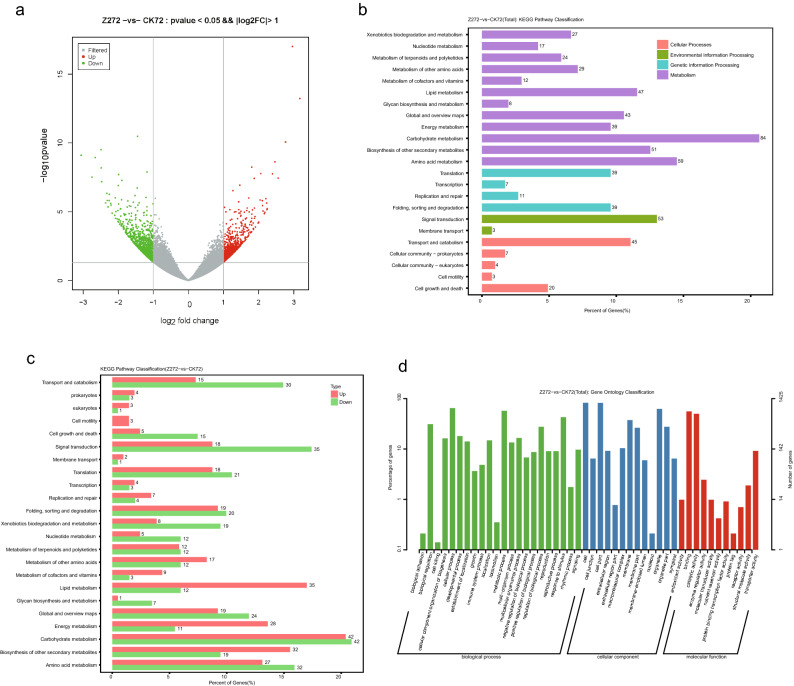
The gene expression of *B. napus* between control (CK72) and HT-COS (Z272) treatments. (**A**) The volcano plot of differentiated expression genes. (**B**) The KEGG pathway enrichment of DEGs. (**C**) The KEGG pathway enrichment of significantly up-regulated genes. (**D**) The Genes Ontology (GO) enrichment of DEGs.

Based on the differential expression gene (DEG) analysis of *B. napus* RNA-seq between HT-COS treatment and control, a total of 1881 DEGs were identified, including 817 significantly up-regulated genes and 1064 significantly down-regulated genes (Fig. 2A). Based on the analysis of gene function, 671 genes were enriched in 23 KEGG pathways, such as carbohydrate metabolism, amino acid metabolism, biosynthesis of other secondary metabolites and signal transduction, and so on (Fig. 2B, Supplementary Figs. 1–3). Compared to the down-regulated genes, the up-regulated genes were significantly enriched in the pathway of lipid metabolism, energy metabolism, and biosynthesis of other secondary metabolites under the HT-COS treatment, indicating that it may provide energy for the growth and development of *B. napus* (Fig. 2C). In addition, there were 1425 DEGs enriched in Genes Ontology (GO), such as growth, metabolic process, response to stimulus, and so on (Fig. 2D).

### Enhanced plant hormone synthesis of *Brassica napus* under the HT-COS treatment

Auxins are mainly composed of indole-3-acetate (IAA), which are one type of plant hormone that is involved in many developmental processes, including cell division, cell differentiation, phototropism, root gravitropism, apical dominance, and vascular differentiation^18^. As a plant growth hormone, IAA plays an important role in regulating plant growth and development^19^.

**Figure 3:**
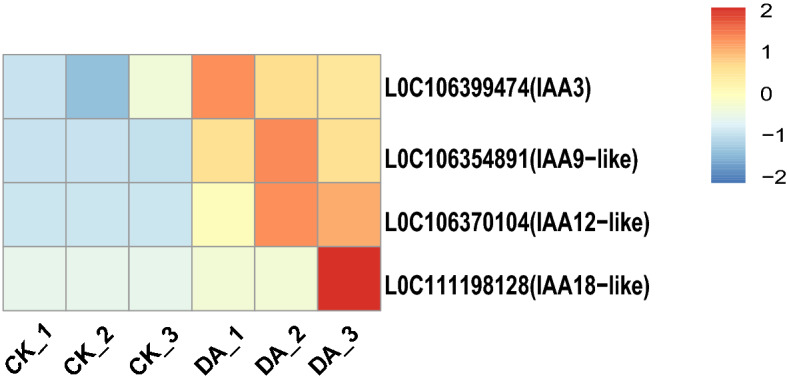
The heat map of plant hormone gene expression in *B. napus* between control (CK) and HT-COS (DA) treatments.

In this study, the content of indole-3-acetate (IAA) under the HT-COS treatment was about 5.0 times higher than that under the control condition (Supplementary Table 1). Based on the analysis of transcriptome data, three AUX/IAA genes (IAA9, IAA12, and IAA3) and one auxin response factor (ARF18) of *B. napus* related to plant hormone signal transduction were significantly up-regulated under the HT-COS treatment (Fig. 3). Furthermore, the plant height, fresh weight, dry weight, and the number of leaves of *B. napus* under the HT-COS treatment were 25.57 ± 0.61 cm, 15.72 ± 0.66 g, 2.18 ± 0.14 g, and 14.2 ± 0.7 respectively, which were significantly higher than that in the control condition (Table 2).

**Table 2:** Effects of HT-COS treatment on *B. napus* growth.

Treatments	Plant height/cm	Fresh weight/g	Dry weight/g	Number of leaves
CK^a^	18.40 ± 0.98	11.68 ± 0.65	1.64 ± 0.04	10.57 ± 0.75
DA treatment^a^	25.57 ± 0.61	15.72 ± 0.66	2.18 ± 0.14	14.23 ± 0.70
Sig	0.000	0.002	0.003	0.003

^a^Data are mean ± standard error. The results of the independent samples t-test showed that the difference between samples was significant when Sig. < 0.05.

### Verification of the transcriptome reliability using qRT-PCR

**Figure 4:**
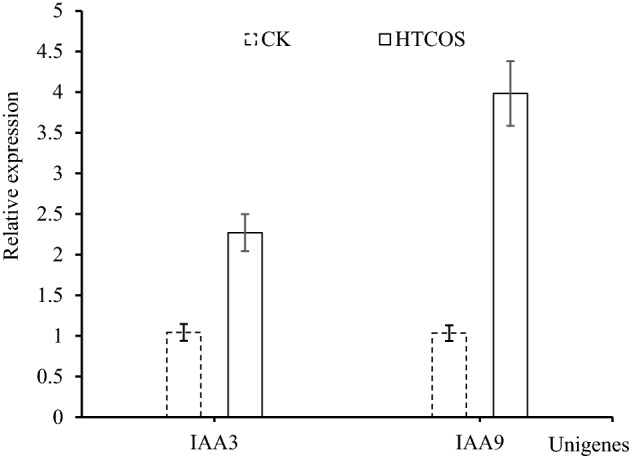
Expression of genes IAA3 and IAA9 relative to control (CK) at the transcriptome level in the HT-COS (DA) treated group and validation by qRT-PCR − ΔΔCt values.

To confirm the differential expression of the DEGs under the HT-COS treatment condition, two IAA genes (IAA3 and IAA9) were selected for qRT-PCR analysis. The change of expression level of the selected DEGs determined by qRT-PCR was consistent well with the result of RNA-Seq analysis (Fig. 4). The primers of DEG were shown in Table 3. These results further demonstrated that under HT-COS treatment, the IAA genes expression were significantly up-regulated more than 2 times.

**Table 3:** List of primers used for the real-time RT-PCR.

Unigene ID	Primer sequence (5′–3′)	Annealing temperature/°C	Fragment size/bp
NC_027771.2: IAA3	F: TGGGACTACCAGGAACAG	55	306
	R: CGACCACCCTCACTATCA		
NC_027767.2: IAA9	F: GGCCCTTCTTACCTTTGG	55	163
	R: TTCCGTGGCACATCCTTC		
ACT7	F: GCTGACCGTATGAGCAAAG	55	182
	R: AAGATGGATGGACCCGAC		

## Discussion and conclusion

Chitosan, which is a good biogenic pesticide, not only induce disease resistance in plants to improve the efficacy of some other pesticides, but also enhance abiotic stress tolerance^20–23^. In addition, it was found that the net photosynthetic rate, stomatal conductance and intercellular CO_2_ concentration of oilseed rape leaves were significantly increased after chitosan spraying on seedling leaves, which was achieved through NO and ABA pathways^24^. In this study, we found that seed dressing with hetero-chitooligosaccharides (HTCOS) can promote the growth of rapeseed, with a 35% increase in plant height, fresh weight, dry weight and the number of leaves compared with that without HTCOS treatment, consistent with previous researches^20,21,23^. IAA is an important plant hormone that regulates many processes of plant growth and development and is closely related to the response of plants to adversity stress. γ-aminobutyric acid is a non-protein amino acid commonly found in animals, plants and microorganisms, and plays an important role in plant growth, development and resistance response. Interestingly, the contents of indole-3-acetate (IAA) and γ-aminobutyric, were significantly upregulated 5.0, and 4.3 times under the HTCOS treatment, respectively. Furthermore, four genes (IAA9, IAA12, IAA3, and ARF18) related to plant hormone signal transduction in *B. napus* were significantly up-regulated under the HT-COS treatment. These results indicated that the HT-COS treatment induced accumulation of plant hormone IAA in the *B. napus*, promoted the up-regulation expression of key genes in the signaling pathway of plant growth, and accelerated the agronomic traits of plant growth, such as plant height, branching number and biomass. Those results suggested that the HT-COS could induce the biosynthesis and hormone signal of plant hormone in rapeseed, promoting the recovery of sugar metabolism levels in leaves to provide the carbon source required for plant growth and development.

Chitin-derived highly deacetylated oligosaccharide COS is an effective immune exciton in plants, inducing resistance to cold, disease, and insects. Today, COS products in practical application usually have a high degree of deacetylation over 85%. In contrast, the plant-acting activity of low-level deacetylated chitin oligosaccharides has been well studied their plant-specific receptors have been identified^25,26^. It has been shown that NACOS with a polymerization degree greater than 3 have stronger plant-inducing activity and better activity compared to COS with the same polymerization degree. It was suggested that the acetyl group of NACOS is essential for the binding of the oligosaccharide to the receptor, resulting the higher activity. However, the solubility of high polymerization degree (DP > 8) NACOS is low in both water and organic solutions, and the large-scale production of NACOS is not available, which limits their wider application.

Recently, an enzyme-based well-controlled production strategy of HTCOS oligosaccharide products with 50–80% deacetylation was established. HTCOS has enough acetyl groups for receptor binding and at the same time shows good water solubility at a high degree of polymerization. The degree and pattern of deacetylation of HTCOS is closer to the fungal cell wall chitin/chitosan, the natural plant excitons, which may also be the structural basis for their excellent plant immune-inducing activity. This study presents the evidence of excellent activity and potential mechanism of hetero-chitooligosaccharides (HTCOS). Our results will provide not only new insights for chitin-derived oligosaccharides in promoting plant growth but also theoretical guidance for future field applications.

## Materials and methods

### Experimental species and reagents

Kale-type oilseed rape Hua You No. 9 was produced by GuchengShengguang Seed Industry Co. Hetero-chitooligosaccharides (HTCOS) were provided by Prof. Yuguang Du at the Institute of Process Engineering, Chinese Academy of Sciences.

### Experimental methods

Full oilseed rape seeds were selected and placed in a 9 cm Petri dish lined with filter paper. 8 ml of sterile water was added and incubated overnight at 4 °C to allow the seeds to absorb the water. The seeds are then placed in an artificial incubator at 26 ± 2 °C for 24 h, selected for consistent germination potential, and planted in a 161-hole floating tray with the seedling substrate to grow till seedlings have 2 leaves and 1 heart. The seedlings are then transferred to a 10 cm diameter, 8.5 cm high seedling bowl with the seedling substrate, and 1 seedling of rape is planted in each pot. When seedling growth reached 4 leaves and 1 heart, potted oilseed rape seedlings at same growth stage were selected and sprayed with 80 mg/L of HTCOS (recorded as DA treatment group), while a clear water treatment was set as a blank control. Each pot was sprayed with 10 mL HTCOS or water, and a total of 42 plants were treated. 12 samples were used for the transcriptomic and metabolomic analyses, and the remaining 30 plants were used for phenotypic data statistics. Treated rape seedlings were placed in an artificial climate chamber at a temperature of 26 ± 2 °C, humidity RH 70 ± 10%, and light L:D = 14:10.

### Sample collection

After 72 h of HTCOS treatment, the third and fourth leaves of each plant, counted from bottom to top, were packed in 50 ml centrifuge tubes and frozen rapidly in liquid nitrogen. Part of the samples was sent to Shanghai Ouyi Biomedical Co Ltd for determination of transcriptome (3 biological replicates) and metabolome (6 biological replicates), and the rest part was kept in an ultra-low temperature refrigerator at − 80 °C.

### Phenotypic measure

After 21 d of HTCOS treatment, the height, number of leaves of seedling, wet weight of above-ground parts, and dry weight of above-ground parts were investigated. Both treatment and control groups include 10 plants and were replicated three times, counting 30 plants for each group.

### Metabolite extraction

360 μL of cold methanol and 40 μL of 2-chloro-l-phenylalanine (0.3 mg/mL) dissolved in methanol as internal standard was added to each sample, samples were placed at – 80 °C for 2 min, and then ground at 60 Hz for 2 min. The mixtures were ultrasonicated at ambient temperature for 30 min. 200 μL of chloroform was added to the samples. The mixtures were vortexed. 400 μL water was added and samples were vortexed again, and then ultrasonicated at ambient temperature for 30 min. The samples were centrifuged at 12,000 rpm for 10 min at 4 °C. QC sample was prepared by mixing same amount of aliquots from all samples. 80 μL of 15 mg/mL methoxylamine hydrochloride in pyridine was subsequently added. The mixture was vortexed vigorously for 2 min and incubated at 37 °C for 90 min. 80 μL of BSTFA (with 1% TMCS) and 20 μL n-hexane were then added into the mixture, which was vortexed vigorously for 2 min and then derivatized at 70 °C for 60 min. The samples were placed at ambient temperature for 30 min before GC–MS analysis.

The derivatized samples were analyzed on an Agilent 7890B gas chromatography system coupled to an Agilent 5977A MSD system (Agilent Technologies Inc., CA, USA). A DB-5MS fused-silica capillary column (30 m × 0.25 mm × 0.25 μm, Agilent J & W Scientific, Folsom, CA, USA) was utilized to separate the derivatives. Helium (> 99.999%) was used as the carrier gas at a constant flow rate of 1 mL/min through the column. The injector temperature was maintained at 260 °C. The initial oven temperature was 60 °C, ramped to 125 °C at a rate of 8 °C/min, to 210 °C at a rate of 4 °C/min, to 270 °C at a rate of 5 °C/min, to 305 °C at a rate of 10 °C/min, and finally held at 305 °C for 3 min. The temperature of MS quadrupole and ion source (electron impact) was set to 150, and 230 °C, respectively. The collision energy was 70 eV. Mass data were acquired in a full-scan mode (50–500 m/z).

### Metabolite analysis

ChemStation (version E.02.02.1431, Agilent, USA) software was used to convert the raw data to CDF format, and then analyzed with Chroma TOF software (version 4.34, LECO, St Joseph, MI) for data processing. Metabolites were annotated through Fiehn or NIST database. After alignment with the Statistic Compare component, the ‘raw data array’ (.cvs) was obtained from raw data with three-dimension data sets including sample information, peak names (or retention time and m/z), and peak intensities. In the ‘data array’, all internal standards and pseudo positive peaks (caused by background noise, column bleed, or BSTFA derivatization procedure) were removed. The data were normalized to the total peak area of each sample, and multiplied by 10,000, and the peaks from the same metabolite were combined.

Data were transformed by log2 (use 0.000001 to replace 0 before transforming), and the resulting data matrix was then imported into the SIMCA software package (v14.0). Principle component analysis (PCA) and (orthogonal) partial least-squares-discriminant analysis (OPLS-DA) was performed to visualize the metabolic difference among experimental groups, after mean centring and unit variance scaling. The Hotelling’s T2 region, shown as an ellipse in score plots of the models, defines the 95% confidence interval of the modeled variation. Variable importance in the projection (VIP) ranks the overall contribution of each variable to the OPLS-DA model, and those variables with VIP > 1 are considered relevant for group discrimination.

The differential metabolites were selected based on both the variable influence on projection (VIP) values with statistically significant threshold from the OPLS-DA model and p-values from a two-tailed Student’s t-test on the normalized peak areas from different groups, where metabolites with VIP values larger than 1.0 and p-values less than 0.05 were considered as differential metabolites.

### RNA extraction and establishment of cDNA library

The total RNA of *B. napus* leaf under the HTCOS conditions was extracted using an RNA extraction kit and the quality of extracted RNA was determined using Nanodrop 2000 spectrophotometer. The integrity of total RNA was checked using formamide denaturing gel electrophoresis, and mRNA was isolated from total RNA using Dynabeads Oligo (dT) 25 isolation beads. The mRNA of the extracted sample was used for building a cDNA library using a reverse transcription kit based on the manufacturer’s instruction (NEBNext Ultra™ RNA Library PrepKit for Illumina). The insert size of the cDNA library was checked by Agilent 2100 bioanalyzer. The cDNA library was sequenced on the Illumina sequencing platform using the paired-end (PE) technology within a single run, in which 150 bp PE reads were obtained.

### Sequencing and differentially expressed genes (DEGs) analysis

The reference genome sequence of *Brassica napus* was downloaded from NCBI (GenBank: GCA_000686985.2). The raw transcriptome data of all samples in this trial were uploaded to the NCBI database (BioProject: PRJNA781006). The cDNA library with high quality was sequenced on the Illumina sequencing platform based on second-generation sequencing technology. To obtain localization information of reads in reference genomic, clean reads were compared with reference genomic using HISAT2-2.0.5^27^, and the expression level was calculated using the FPKM method (fragments per kilobase million)*.* The difference expressed genes (DEGs) were analyzed using the DEseq2 package version 3.8.6^28^. The genes with|log_2_ Fold Change|≥ 1, and false discovery rate (FDR) < 0.05 were considered as differentially expressed genes (DEGs). The KEGG enrichment analysis of functional significance terms based on KEGG (http://www.kegg.jp/kegg/pathway/html) database was conducted using a hypergeometric test to find significant KEGG terms in DEGs for comparison with the genome background.

### Validation of gene expression by qRT-PCR

To verify genes that were differentially expressed in HTCOS-treated samples compared with the control, qRT-PCR was performed, using an iQ SYBR Green SuperMix kit (Bio-Rad) on an iCycleriQ system (Bio-Rad, Hercules, CA, USA). Gene-specific primers of 3 candidate genes (Table 3) were designed using the Primer Premier 5.0 software. For qRT-PCR, BnACTIN (ACT7) is used as reference genes^29,30^. The gene encoding actin, which displays a stable expression under different stress condition, was used as an internal control for data normalization^31^. For each sample, first-strand cDNA was synthesized from 1 μg from the pooled RNA sample of the CK or HTCOS plants, using a Revert Aid First-Strand cDNA Synthesis Kit (ThermoScientific, Fermentas, Vilnius, Lithuania), according to the manufacturer’s instructions. All reactions were performed in triplicate with six replicates. Expression levels of each gene are presented as the fold change relative to that of the control gene, calculated with the ${2}^{-\Delta \Delta \mathrm{Ct}}$ method^32^.

### Research involving plants

This paper does not involve animal and human related studies, and the experimental methods are in accordance with all the relevant guidelines and regulations. Kale-type oilseed rape Hua You No. 9 was produced by GuchengShengguang Seed Industry Co.

## Data availability 

Supplementary Table 1.xls contains the levels of 26 metabolites in *B. napus* were significantly changed under the hetero-chitooligosaccharide treatment. The reference genome sequence of *B. napus* was downloaded from NCBI (GenBank: GCA_000686985.2). The raw transcriptome data of all samples in this trial were uploaded to the NCBI database (BioProject: PRJNA781006). In addition to that, the authors affirm that all data necessary for confirming the conclusions of the article are present within the article, figures, and tables.

## Supplementary Information

Supplementary Information 1.

Supplementary Information 2.

Supplementary Information 3.

Supplementary Information 4.
