# Supplementary material for: Metabolomics and transcriptomics reveal the effect of hetero-chitooligosaccharides in promoting growth of Brassica napus
Source: Sci Rep. 2022 Dec 8;12:21197. doi: 10.1038/s41598-022-25850-7 (PMC9731942; doi:10.1038/s41598-022-25850-7)
Supplement: Supplementary file 2 — Supplementary Information 2. [file 41598_2022_25850_MOESM2_ESM.pdf]

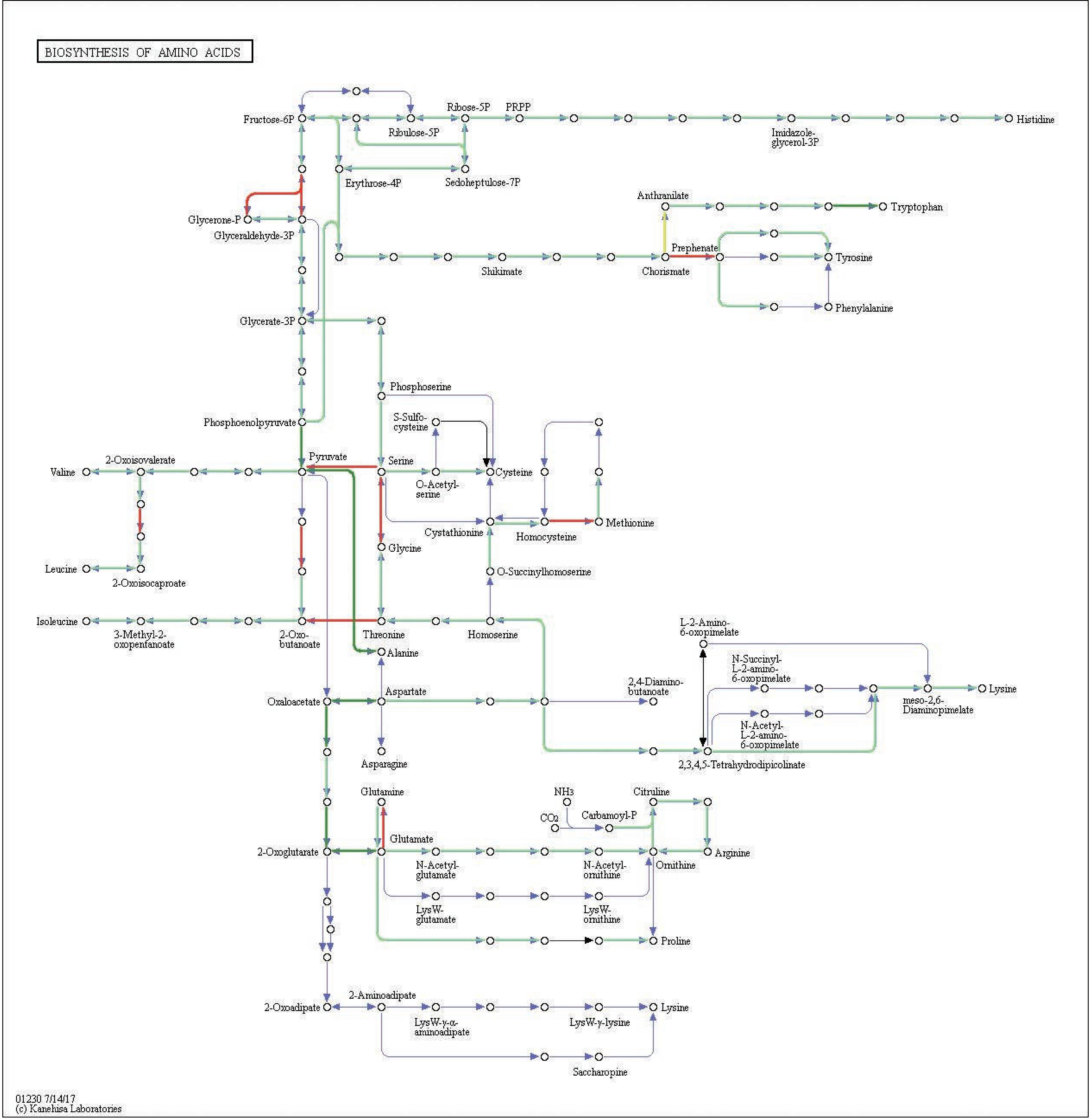

Supplementary Figure 2. The KEGG pathway of Amino acids biosynthesis. The green lines show significantly downregulated genes. The red lines show genes that are significantly upregulated.
