# Supplementary material for: Metabolomics and transcriptomics reveal the effect of hetero-chitooligosaccharides in promoting growth of Brassica napus
Source: Sci Rep. 2022 Dec 8;12:21197. doi: 10.1038/s41598-022-25850-7 (PMC9731942; doi:10.1038/s41598-022-25850-7)
Supplement: Supplementary file 3 — Supplementary Information 3. [file 41598_2022_25850_MOESM3_ESM.pdf]

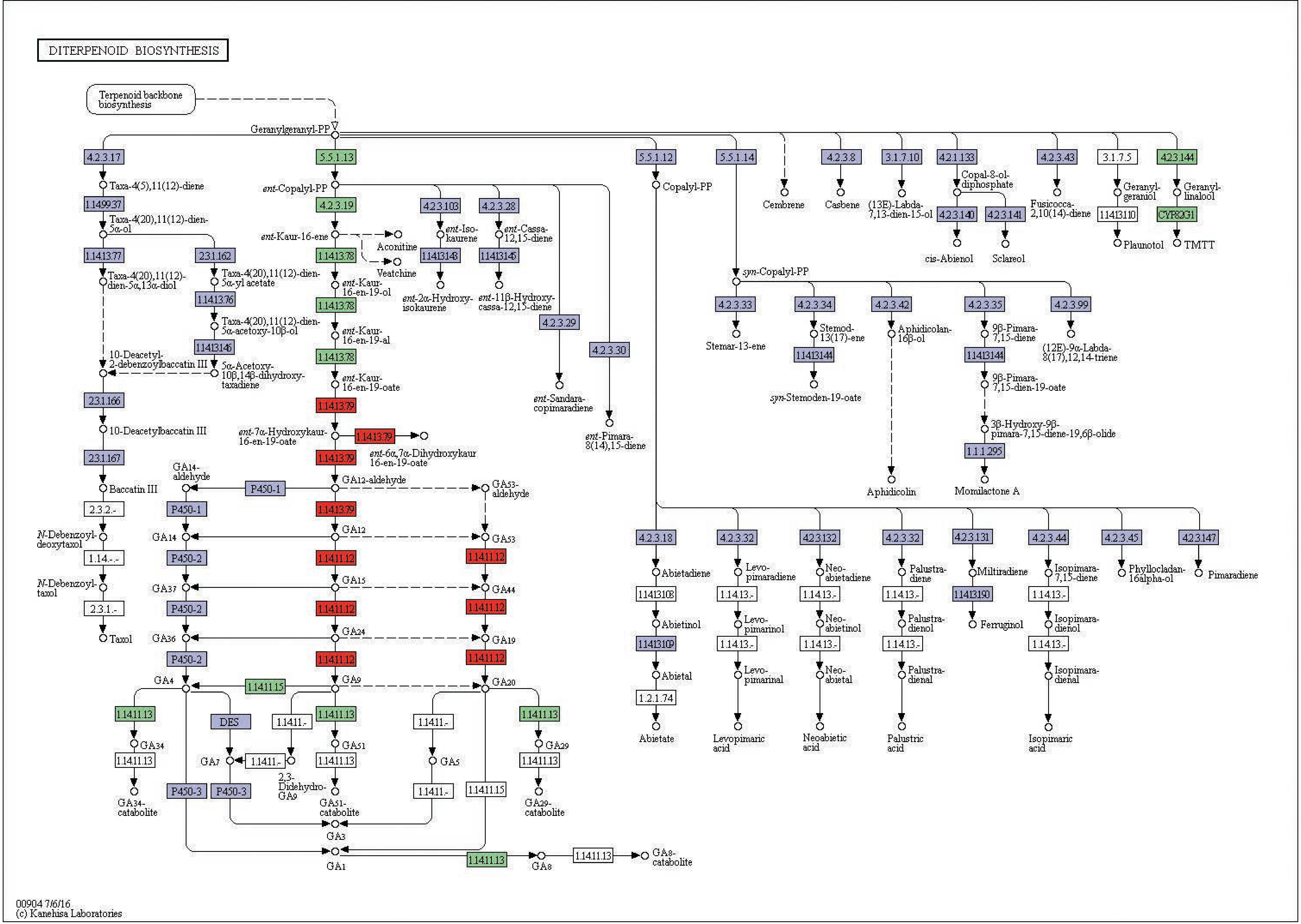

Supplementary Figure 3. The KEGG pathway of diterpenoid biosynthesis. The green boxes show significantly downregulated genes. The red boxes show genes that are significantly upregulated.
